# Serum Levels of Gelatinase Associated Lipocalin as Indicator of the Inflammatory Status in Coronary Artery Disease

**DOI:** 10.1155/2012/189797

**Published:** 2012-09-04

**Authors:** Nikolaos Kafkas, Christos Demponeras, Filitsa Zoubouloglou, Loukia Spanou, Dimitrios Babalis, Konstantinos Makris

**Affiliations:** ^1^Cardiology Department, KAT General Hospital, Athens, Greece; ^2^Clinical Biochemistry Department, KAT General Hospital, Athens, Greece

## Abstract

*Background*. Atherosclerosis is a chronic inflammatory disease and the acute clinical manifestations represent acute on chronic inflammation. Neutrophil gelatinase-associated lipocalin (NGAL) is found in the granules of human neutrophils, with many diverse functions. The aim of this study was to evaluate the hypothesis that levels NGAL in blood may reflect the inflammatory process in various stages of coronary artery disease. *Methods*. We studied 140 patients, with SA 40, UA 35, NSTEMI 40, and STEMI 25, and 20 healthy controls. Serum NGAL was measured upon admission and before coronary angiography. *Results*. Significant differences were observed in median serum-NGAL(ng/mL) between patients with SA (79.23 (IQR, 37.50–100.32)), when compared with UA (108.00 (68.34–177.59)), NSTEMI (166.49 (109.24–247.20)), and STEMI (178.63 (111.18–305.92)) patients and controls (50.31 (44.30–69.78)) with significant incremental value from SA to STEMI. We observed a positive and significant correlation between serum-NGAL and hs-CRP (spearman coefficient rho = 0.685, *P* < 0.0001) as well as with neutrophil counts (*r* = 0.511, *P* < 0.0001). *Conclusions*. In patients with coronary artery disease serum levels of NGAL increase and reflect the degree of inflammatory process. In patients with acute coronary syndromes, serum levels of NGAL have high negative predictive value and reflecting the inflammatory status could show the severity of coronary clinical syndrome.

## 1. Introduction

Systemic inflammation participates in atherosclerosis evolution from the early development of endothelial dysfunction, to formation of mature atheromatic plaques, to the ultimate endpoint, rupture, and thrombotic complications [1]. Plaque rupture with the formation of an occlusive thrombus is the cause of acute coronary syndromes (ACS) [2]. Inflammatory cells, involving activated neutrophils, are more frequently found in plaques vulnerable to rupture [3]. Neutrophil activation has been reported in unstable angina (UA) and acute myocardial infarction (AMI) but not in patients with stable angina (SA) [4–10]. This activation seems to precede myocardial injury in patients with AMI [11]. Therefore biomarkers of neutrophil activation could be of prognostic and even diagnostic importance. 

Recent studies have shown that gelatinase B also known as matrix metalloproteinase-9 (MMP-9), an endopeptidase capable of degrading the extracellular matrix, is thought to be associated with atherosclerosis, and plaque rupture [12, 13]. Therefore, MMP-9 is considered to be an important mediator of vascular remodeling and plaque instability. The MMP-9 action is enhanced b neutrophil gelatinase-associated lipocalin (NGAL), also known as lipocalin-2, a 25 kDa glycoprotein, that is, found in the granules of human neutrophils, with many diverse functions, such as scavenger of bacterial products, modulator of inflammation, iron trafficking, and apoptosis [14]. 

The formation of a complex with NGAL and MMP-9 is crucial for atherosclerotic plaque erosion and thrombus formation [15]. NGAL is also produced by kidney tubular cells in response to various ischemic or toxic insults and has been proposed as an early biomarker for the diagnosis of acute kidney injury [16, 17]. 

In this study, we hypothesized that levels NGAL in blood may reflect the extent of neutrophil activation in various stages of ACS and could discriminate various types of ACS (UA, NSTEMI, and STEMI) and stable from unstable coronary syndromes.

## 2. Methods

### 2.1. Study Design and Population

One hundred and seventy consecutive patients programmed for coronary angiography to the Invasive Cardiology Department of the KAT General Hospital Athens, Greece, were recruited for this study, from June 2010 to October 2010. 

The study was performed according to the principles of the Declaration of Helsinki and was approved by the hospital's ethics committee. Written informed consent was obtained from all participating patients.

Thirty patients were excluded from the study. Exclusion criteria included a negative coronary angiography in patients with a typical chest pain which was considered as angina or had a false positive single photon emission computed tomography (SPECT), any surgery in the previous six months, liver disease, end stage renal disease, renal cardiac or liver transplantation, neoplasia, and infection since all these can affect serum-NGAL levels. 

**Figure 1:**
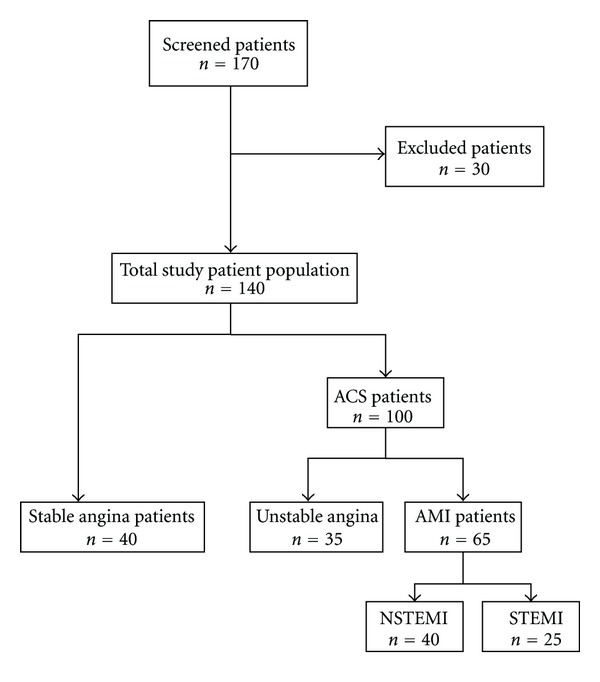
Flow diagram of subject recruitment. Ten patients were excluded for the following reasons: malignant diseases (*n* = 1), active infections (*n* = 3), and end stage renal disease (*n* = 3) recent surgery (*n* = 3). Also 20 patients with negative coronary angiography were excluded.

The 140 patients who fulfilled the study criteria after the clinical assessment and final diagnosis were divided into the following 4 groups: SA (*n* = 40), UA (*n* = 35), NSTEMI (*n* = 40), and STEMI (*n* = 25). Twenty (20) healthy amateur athletes without risk factors served as control group (Figure 1). The demographics and clinical characteristics of patients and controls are shown on Table 1.

### 2.2. Clinical Assessment

 All patients, upon presentation in emergency room, underwent an initial clinical assessment that included clinical history, physical examination, 12-lead ECG, continuous ECG monitoring, and standard blood tests (including white blood cell, polymorphonuclear neutrophil counts, and troponin-I). These tests were repeated at 6 at 12 and 24 hours as long as clinically indicated. To determine the final diagnosis for each patient 2 cardiologists blinded to NGAL results reviewed all patients available records (including patient history, laboratory results, radiologic testing, ECG, echocardiography, and coronary angiography) at the completion of their hospital stay.

The SA group consisted of patients with angiographically documented organic coronary stenosis >70% by quantitative coronary angiography in major arteries who had chronic symptoms of angina or a positive SPECT test. UA was diagnosed in patients with typical angina at rest, or a sudden increase in episodes of a previously stable angina.

AMI was diagnosed when there was evidence of myocardial necrosis in a clinical setting consistent with myocardial ischemia. Necrosis was diagnosed by a rising and/or falling pattern of troponin-I with at least one value above the cutoff value (defined as the 99th percentile of a normal population where the assay shows an imprecision <10%). Our troponin-I assay fulfills the imprecision criteria for concentration >0.2 ng/mL.

### 2.3. Sample Collection

 Serum and K_2_EDTA-plasma samples were collected from all SA patients in the morning before the coronary angiography. In all ACS patients PCI was performed within 24 hours from admission and the blood samples were collected on admission. From all healthy subjects, samples were collected in the morning and before training. Serum samples were kept frozen at −80°C until tested. 

### 2.4. Laboratory Tests

Total white blood cell count (WBC), and peripheral polymorphonuclear neutrophil count (PMN) were assessed using the Cell-Dyn Sapphire haematology analyzer (Abbott, Chicago, Il, USA). Serum creatinine was measured with a modified jaffe method on Architect ci16200 analyzer (Abbott, Chicago, Il, USA). High-sensitivity CRP (hs-CRP) was measured with a turbidimetric assay on the same analyzer. Troponin-I was measured with a chemiluminescent immunoassay on the same analyzer. Serum-NGAL was measured with an ELISA (Bioporto, Gentofte, Denmark). 

### 2.5. Statistical Analysis

This was performed with NCSS statistical program. Normality of distributions for quantitative data was tested with the Shapiro-Wilk test. For normally distributed parameters data were presented as means + standard deviation. For comparisons of means among patients groups and healthy controls a standard one-way anova test was used. Because serum-NGAL did not distribute normally, nonparametric tests were used: the Mann–Whitney test and the Kruskal-Wallis test with multiple-comparison procedures (Dunn's method) for comparisons between groups, and the Friedman test and the Wilcoxon test with the Bonferroni correction for comparisons within groups. Correlations were determined with use of Spearman's rank-correlation coefficient. Chi-square statistics were used for categorical variables. A *P* value of less than 0.05 (two-tailed) was considered to indicate statistical significance. Data are reported as medians and interquartile ranges. Receiver-operating characteristic (ROC) analysis was performed to calculate the area under the curve (AUC) and define cutoff points. Cutoff values were chosen after cost-benefit analysis. Differences between AUCs were investigated with the non-parametric approach of DeLong, DeLong and Clarke-Pearson. Statistics were performed with the NCSS 2004 Statistical and Power Analysis Software (NCSS Inc, Kaysville, UT, USA).

## 3. Results

The mean age and the mean BMI of the patients did not differ significantly among the four groups whereas the controls were significantly younger and their BMI was significantly lower (ANOVA-test). The proportion of diabetic patients did not differ significantly among four patient groups (chi-square = 1.69, *P* = 0.639) as well as the proportion of patients with hypertension and dyslipidemia (chi-square = 1.63, *P* = 0.652). Finally smoking habits did not differ significantly in the first three patient groups while it was significantly higher in group 4. Those risk factors were absent from our controls.

### 3.1. Levels of Serum-NGAL in Patients with CAD and Controls

**Figure 2:**
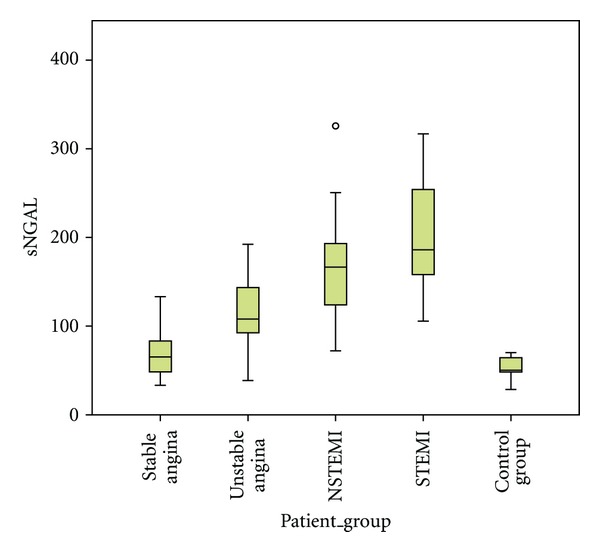
Box plots of median serum-NGAL values (ng/mL) among the 4 patients groups and healthy controls (*y* axis is in linear scale).

The differences we observed in the median serum-NGAL values among the 4 patient groups and the healthy controls were significant (*P* < 0.001, ANOVA test). Further statistical analysis using multiple-comparisons between groups revealed that the median serum-NGAL levels in UA (108.00 ng/mL), NSTEMI (166.49 ng/mL), and STEMI (178.63 ng/mL) patients were significantly higher than in patients with SA (79.23 ng/mL) and healthy controls (50.31 ng/mL) (Table 2 and Figure 2). Also significant were the differences observed between healthy controls and SA patients, between UA patients and patients with AMI (NSTEMI or STEMI). Patients with STEMI had higher levels of NGAL than patients with NSTEMI but the difference was nonsignificant (Table 2).

### 3.2. Correlation between Levels of NGAL and Inflammatory Markers

The median plasma levels of hs-CRP were similar in patients with SA (0.40 mg/dL) and those with UA (0.69 mg/dL) and were significantly higher than the levels in the control group (0.12 mg/dL). Hs-CRP levels were significantly increased in patients with NSTEMI (1.20 mg/dL) and STEMI (6.76 mg/dL).

**Figure 3:**
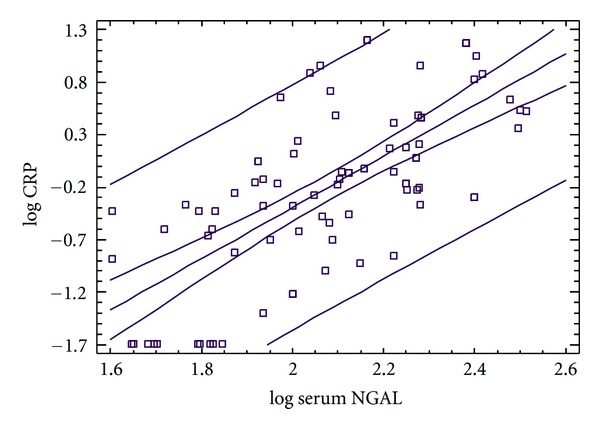
Regression analysis between serum-NGAL and hs-CRP.

In order to further investigate the relationship between serum-NGAL and hs-CRP, we performed regression analysis between serum-NGAL and hs-CRP (Figure 3). This analysis revealed that there is a linear and positive correlation between hs-CRP and serum NGAL (spearman rank correlation coefficient rho = 0.685, *P* < 0.0001).

**Figure 4:**
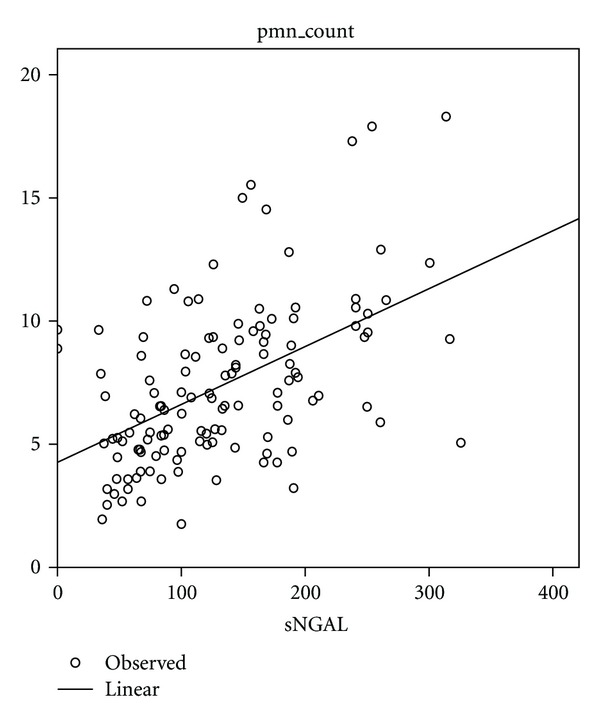
Scater plots of PMN values versus serum NGAL.

The differences that were observed among the four patient groups in WBC and PMN counts were statistically significant (*P* < 0.001, ANOVA-test). There was a positive and significant correlation between serum-NGAL and WBC (*r* = 0.510, *P *< 0.0001) and PMN (*r* = 0.511, *P* < 0.0001) counts (Figure 4).

In a multivariate regression analysis model entering as independent parameters age, serum creatinine, hs-CRP, and PMN count, we identified only hs-CRP (*P* < 0.005) and PMN count (*P* < 0.0001) as independent predictors of serum-NGAL levels.

### 3.3. The Diagnostic Value of NGAL in Discriminating Stable from Unstable Patients

**Figure 5:**
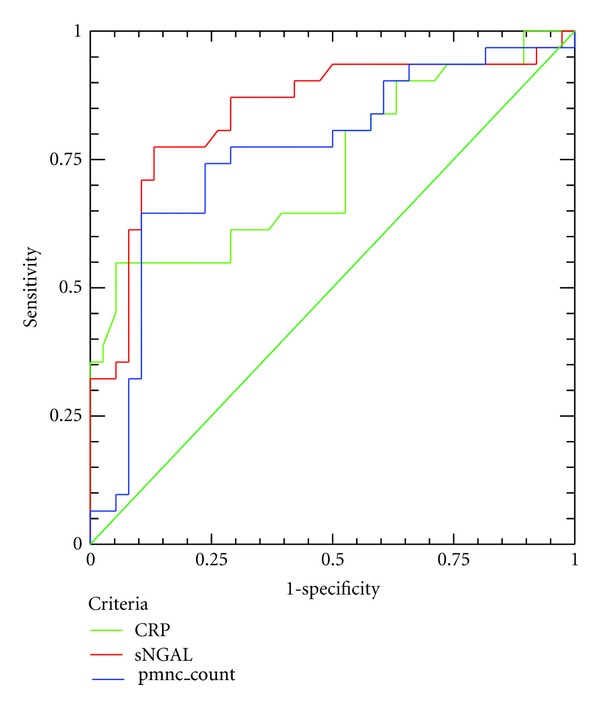
ROC curve analysis for CRP (green line), serum-NGAL (red line), and PMN count (blue line) for the discrimination of UA patients from patients with SA.

**Figure 6:**
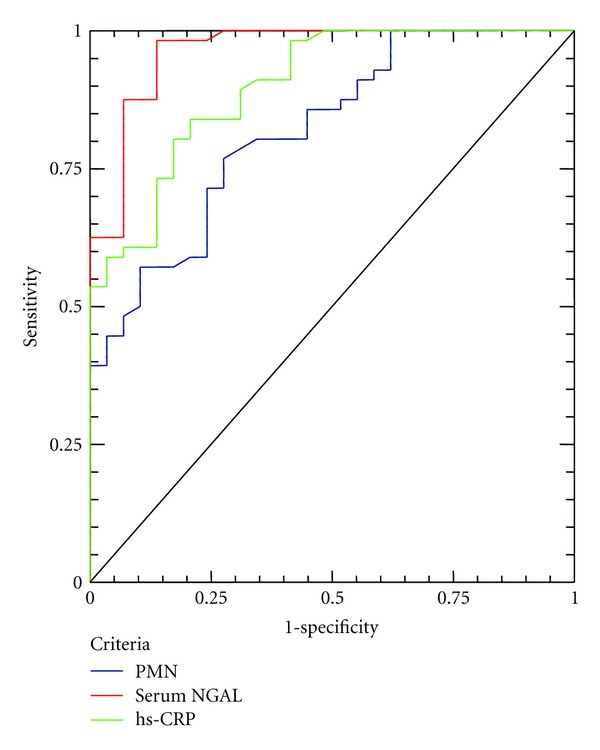
ROC curve analysis for CRP (green line), serum-NGAL (red line), and PMN (blue line) for the discrimination of ACS patients from patients with SA.

ROC curves were generated for sensitivity and specificity with the respective areas under the curve for serum-NGAL hs-CRP and PMN counts (Figures 5 and 6).

The diagnostic value for serum-NGAL in discriminating patients with UA, from those with SA is high (AUC = 0.852) and better than of hs-CRP (AUC = 0.735) or PMN count (AUC = 0.761). If we use as cutoff for serum-NGAL 83.74 ng/mL, we can predict an UA event with sensitivity and specificity, 82.8% and 75%, respectively. The negative predictive value of this cutoff is high (97.28%). 

The diagnostic value for serum-NGAL in discriminating ACS patients, from patients with SA is high (AUC = 0.929) and better than of hs-CRP (AUC = 0.794) and PMN count (AUC = 0.830). If we use as cut-off for serum-NGAL 89.29 ng/mL, we can discriminate an ACS patient from a stable patient with sensitivity and specificity, 89.3% and 81.6%, respectively. The negative predictive value of this cutoff is high (98.65%).

## 4. Discussion

In this study, we demonstrated that serum levels of NGAL are higher in patients with CAD than in healthy controls patients. Among ACS patients, these levels are gradually elevated according to the severity of the coronary clinical syndrome (UA, NSTEMI, and STEMI). Also serum levels of NGAL are higher in patients with ACS than in patients with SA and could be used, with high negative value, to discriminate patients with stable or unstable coronary syndromes. 

The relevance of NGAL to cardiovascular disease (CVD) remains primarily unknown. Elevated plasma NGAL levels were associated with atherosclerosis and were implicated as a predictor for cardiovascular mortality after cerebrovascular ischemia, possibly because of activation of blood leukocytes [18–20]. Although in recent reports has been shown that NGAL is present in atherosclerotic plaques and in human abdominal aortic aneurisms, raising the possibility that expression of NGAL can be induced in vascular cells during atherogenesis, the underlying mechanism for the induction of NGAL in vascular cells remains unknown [15, 21]. In further analysis the main source of NGAL was found to be neutrophils, probably recruited in the vascular wall by platelet activation [21]. 

NGAL is considered to have a protective effect on MMP-9 and enhancing its proteolytic activity, could be considered as an important factor indirectly contributing to the progression of aneurism as well as involved in the physiologic and pathologic remodeling of vessel walls. This view is further supported by the observation that similar neutrophil NGAL/MMP-9 overexpression can be found in atherosclerotic plaques, particularly those with intramural haemorrhagic debris and central necrosis [15, 22]. The above evidence supports the clinical observations that high-circulating leucocyte (particularly neutrophil) counts are independent predictors of recurrent ischaemic attacks. This may be explained by their presence in the necrotic core of unstable plaques and by their proteolytic activity towards atherosclerotic tissue and secondary mobilization of thromboembolic fragments [23]. The evidence derived from these experimental studies, showing the close link between neutrophils, their products and the natural history of atherosclerosis, and its complications, generated clinical studies that investigated the clinical utility of serum-NGAL measurements. 

In two recent studies it was found that serum levels of NGAL were significantly elevated in patients with angiographically confirmed CAD compared to those with normal arteries or controls [24, 25]. Our data agree with these reports since we found that levels of serum-NGAL are significantly higher in patients with all clinical syndromes of CAD than in healthy controls, reinforcing the utility of NGAL as biomarker of detection and the extent of CAD. The expression of NGAL from vascular cells during atherogenesis can also explain the differences between patients with SA and control subjects with no risk factors observed in our study. In addition to its induction in the vessels after mechanistic injury, previous studies suggest that NGAL is strongly upregulated in atherosclerotic lesions and also in the heart after ischemic injury [15]. It is possible that NGAL produced by vascular cells could also be secreted into the systemic circulation. 

Inflammation plays a critical role not only in development and progression of atherosclerosis but also in pathogenesis of the destabilization of atherosclerotic plaque that leads to ACS [1, 26]. Activation and degranulation of polymorphonuclear neutrophils and probably an underestimated critical components of an acute coronary inflammation event. Infiltrating macrophages and neutrophils participate in the transformation of stable coronary artery plaques to unstable lesions with a thin fibrous cap [27]. It has been repeatedly reported that thrombosed plaques were densely infiltrated by neutrophils and macrophages [28, 29]. Macrophages and neutrophils and some other types of leukocytes produce various proteolytic enzymes which facilitate the rupture of plaques by thinning and weakening their normally thick and firm cap [30, 31]. NGAL is one protein, that is, produced not only by the distressed kidney but also by activated neutrophils and by the vascular wall cells. Recent studies have shown that neutrophils are the main source of NGAL in blood [32, 33].

Increase in serum NGAL resulting from activation of neutrophils may reflect an acute systemic inflammatory response to events such as stroke, renal failure, or infection [18, 34–36] but are also linked with the presence of chronic inflammatory diseases such as atherosclerosis [18] whose acute clinical manifestations represent acute on chronic inflammation. Besides neutrophils, NGAL is also expressed by epithelial cells, renal tubular cells, and hepatocytes during inflammation or injury [37–39]. Our data agree with the above studies since we found a positive correlation between levels of serum-NGAL and systemic inflammation (expressed by the serum hs-CRP levels and neutrophil count), and also serum levels of NGAL were higher in patients with ACS than with SA. The higher levels of serum-NGAL observed in patients with ACS compared to SA could be explained by the fact that neutrophil activation is present only in patients with acute coronary events (10,11). Also, our results, as far as patients with SA and AMI, are similar with the findings of a recent published study which showed that the plasma level of NGAL is higher in patients with AMI compared with the patients with stable CAD [40].

In clinical practice, levels of serum-NGAL have a high negative predictive value, 97.28% and 98.65% for patients with UA and ACS, respectively. So, serum-NGAL could be used in discriminating of patients with ACS or especially UA from whom with SA or without CAD, giving the possibility to exclude patients with symptoms similar to angina but not having true ACS. As far as the gradual increase of serum-NGAL, according to the seriousness of unstable coronary clinical syndrome, this could reflect the intensity of the inflammatory reaction, as it is expressed by the incremental increase of hs-CRP and neutrophil count and their combination with serum NGAL. Especially between serum-NGAL and hs-CRP, the correlation is linear and positive.

In conclusion, our study shows that serum levels of NGAL increase in patients with CAD with every coronary clinical syndrome and reflect the inflammatory status in the same population. Having high negative predictive value could be used as a marker for the discrimination of SA or chest pain without CAD from those with ACS. Also in patients with ACS, serum levels of NGAL reflecting the inflammatory status could show the severity of coronary clinical syndrome (UA, NSTEMI, and STEMI).

## Figures and Tables

**Table 1:** Patient demographics and clinical characteristics.

Nr	Stable angina	Unstable angina	NSTEMI	STEMI	Control group
	40	35	40	25	20
Sex (male/female)	(31/9)	(27/8)	(33/7)	(20/5)	(16/4)
Age mean (SD)	63.8 (9.85)	64.6 (8.17)	64.5 (9.96)	64.2 (11.19)	41.5 (7.96)
BMI mean (SD)	29.76 (4.89)	28.26 (4.81)	28.65 (3.56)	26.26 (2.12)	23.58 (1.80)
Diabetes *N* (%)	16 (40.00)	14 (40.00)	17 (42.50)	12 (48.00)	0
Hypertension *N* (%)	27 (65.71)	25 (71.43)	20 (70.00)	19 (76.00)	0
Dyslipidemia *N* (%)	27 (67.50)	19 (57.15)	25 (62.50)	17 (68.00)	0
Smoking	12 active 6 quit	17 active 1quit	18 active	14 active 4 quit	0

**Table 2:** Median and range of serum, urine NGAL, hs-CRP serum creatinine, and eGFR(MDRD) among patient groups and healthy controls.

Nr		Stable angina	Unstable Angina	NSTEMI	STEMI	Control group
	Units	40	35	40	25	20
hs-CRP median (*quartiles*)	mg/dL	0.40 (0.05–0.87)	0.69 (0.11–3.69)	1.17 (0.24–13.21)	3.91 (0.31–13.62)	0.12 (0.02–0.25)
WBC count, mean (SD)	(×10^3^)	7.67 (1.99)	9.38 (2.32)	11.50 (2.65)	14.67 (4.92)	nd
Neutrophil count, mean (SD)	(×10^3^)	4.90 (1.72)	6.44 (1.96)	8.27 (2.44)	11.08 (4.15)	nd
s-NGAL (∗) median (*quartiles*)	ng/mL	79.23 (37.50–100.32)	108.00 (68.34–177.59)	166.49 (109.24–247.20)	178.63 (111.18–305.92)	50.31 (44.30–69.78)
s-Creatinine, mean (SD)	*μ*mol/L	79.72 (16.94)	79.86 (17.78)	86.04 (21.74)	88.65 (34.46)	81.26 (7.97)
eGFR(MDRD), mean (SD)	mL/min	89.71 (19.85)	89.63 (22.96)	85.79 (22.20)	81.75 (21.06)	97.65 (19.55)

(∗) *P* < 0.05 for the comparison between patients with SA and controls, *P* < 0.005 for the comparison between patients with SA with patients with UA, and *P* < 0.001 for the comparisons of rest groups, and *P*  =  NS for the comparison of patients with STEMI with NSTEMI.

## Abbreviations and Acronyms

ACS: Acute coronary syndrome
CAD: Coronary artery disease
SA: Stable angina
UA: Unstable angina
AMI: Acute myocardial infarction
NGAL: Neutrophil gelatinase associated lipocalin
NSTEMI: Non-ST-elevation myocardial infarction
STEMI: ST-elevation myocardial infarction
PCI: Percutaneous coronary intervention.
